# Impact of Ventilation Tubes on Pediatric Otitis Media Outcomes: A Retrospective Study From Saudi Arabia

**DOI:** 10.7759/cureus.82434

**Published:** 2025-04-17

**Authors:** Muhanna A Alhusayni, Reem A Alotaibi, Naif H AlSufyani, Razan M Althumali, Abrar A Alzahrani, Majed M Alotaibi, Maram A Alameen, Mohamed M Allam, Marwan F Alnofaie

**Affiliations:** 1 College of Medicine, Taif University, Taif, SAU; 2 Otorhinolaryngology, King Faisal Medical Complex, Taif, SAU

**Keywords:** otitis media, pediatric patients, surgical interventions, tympanometry, ventilation tubes

## Abstract

Introduction: Acute otitis media (AOM) and otitis media with effusion (OME) are common in children, often leading to hearing loss and developmental delay. Ventilation tubes (VTs) manage persistent middle ear effusions by draining fluid and reducing pressure, which can decrease AOM recurrence.

Methodology: This retrospective cohort study reviewed the medical records of 388 pediatric patients at King Faisal Medical Complex, Saudi Arabia, from August 2021 to February 2024. Data on demographics, tympanometry results, treatment outcomes, and surgical interventions were collected. The data was analyzed using IBM SPSS Statistics for Windows, Version 26 (Released 2019; IBM Corp., Armonk, New York, United States). Categorical variables were reported as numbers and percentages, with descriptive statistics applied.

Results: Among 388 children aged 2 to 12 years, girls predominated (56.2%). Tympanometry revealed bilateral type B in 64.4% and unilateral type B in 35.6%. After three months of observation and treatment, 47.6% were relieved, while 52.4% had persistent type B. Most cases were newly diagnosed (95.07%). Adenoid enlargement was observed in 86.70%, allergic rhinitis in 92.61%, and adeno-tonsillar enlargement in 68.47%. Surgical interventions included myringotomy only (15.27%), myringotomy with Shah tube (46.31%), and Shepard tube (38.42%). Complications were rare, with 88.18% achieving complete symptom relief and 4.93% partial relief during postoperative follow-up at 2 weeks, 3 months, 6 months, and 12 months.

Conclusion: VT effectively manages AOM and OME in children, with high rates of symptom relief. However, careful consideration of risks and follow-up is essential to optimize outcomes.

## Introduction

Acute otitis media (AOM) is one of the most common illnesses in children. Although a considerable portion of children experience recurrent AOM (rAOM), defined as three or more occurrences in a six-month period or four or more in a year [1]. Otitis media with effusion (OME), a frequent occurrence in young children, is characterized by the presence of fluid in the middle ear. Hearing loss is a prominent symptom of OME, resulting from reduced mobility of the tympanic membrane and impaired sound conduction [2]. Early childhood middle ear effusion (MEE) may have an impact on motor development [3,4]. Moreover, it reduces the tympanic membrane's mobility, which may postpone language development [5,6] and cause mild to moderate conductive hearing loss [7]. OME and AOM are tightly associated [8]. Children with OME are susceptible to rAOM [9]. OME and developmental consequences connected to hearing loss are also burdened by rAOM [10]. Ventilation tubes (VTs) are one of the most common therapeutics for the treatment of multiple middle ear pathologies, such as persistent MEE, rAOM, or middle ear infections [11]. A VT is a small plastic tube that is inserted into the eardrum to enhance middle ear ventilation and drain fluid. The procedure involves making an incision in the eardrum, removing fluid, and placing the tube. Over time, the tubes naturally move out into the ear canal, and the eardrum closes. VT can alleviate the severity of rAOM by facilitating fluid drainage and relieving pressure on the eardrum. They also enable the administration of localized antibiotic treatment for rAOM.

Prolonged MEE is often managed with VT, and short-term improvements in hearing have been observed without significant long-term effects. However, potential complications of VT include persistent eardrum perforation, conductive hearing loss, tube misplacement, otorrhea, and myringosclerosis, which may result in mild hearing loss. VT is a temporary solution for middle ear problems [12], and the specific type of tube used can vary [13]. After the surgery, it is commonly recommended to ensure that a child's ears remain dry during the immediate postoperative period. Depending on the surgeon's approach, some may allow swimming or bathing after the initial period, while others advise continued precautions to prevent water exposure [14]. The surgical approach involving VTs with adenoidectomy significantly decreases the recurrence of AOM within a year compared to the non-surgical approach (the watchful waiting approach). Children who underwent surgery required considerably fewer AOM-related visits compared to those who did not [15]. The study aimed to investigate the effect of VT insertion in children under 12 years old, focusing on its potential to restore normal hearing. We aim to evaluate the safety and efficacy of VT insertion in pediatric patients, identify predictive factors for symptom recurrence, and assess the impacts of VT type and effusion composition on extrusion rates and complications. Additionally, we seek to evaluate the factors influencing VT extrusion, recurrence rates, and associated complications.

## Materials and methods

Study design, inclusion criteria, and exclusion criteria

We conducted a retrospective cohort study among all pediatric patients presenting to the Otorhinolaryngology Clinic at King Faisal Medical Complex (KFMC) who were diagnosed with OME between August 2021 and February 2024 in Taif, Saudi Arabia. All pediatric patients were included in this study, and there were no exclusion criteria.

Ethical considerations

Prior to conducting the study, ethical approval was obtained from the Scientific Research Ethics Committee at KFMC in Taif, Saudi Arabia (approval number: 2024-E-32). Data confidentiality was maintained throughout the study and after its completion.

Data collection

Data collection was performed retrospectively by reviewing the medical records of patients diagnosed with OME. The data collection sheet included patient demographics, pre-VT insertion audiological measurements, details of the procedure, and subsequent follow-ups.

Data analysis plan

The data was extracted and organized in an Excel sheet (Microsoft Corporation, Redmond, USA). Statistical analysis was then performed using IBM SPSS Statistics for Windows, Version 26 (Released 2019; IBM Corp., Armonk, New York, United States). Since all variables are categorical, they are reported in the form of numbers and percentages. Descriptive statistics were used for all analyses.

## Results

Our study included 388 children from KFMC, Taif, Saudi Arabia, from August 2021 to February 2024. Notably, gender distribution showed more females (n=218, 56.2%) than males (n=170, 43.3%) with an age range of 2 to 12 years. Tympanometry results indicated that a majority of the children had bilateral type B tympanogram patterns (n=250, 64.4%), while unilateral type B was present in 138 children (35.6%). After three months of watchful waiting with follow-up and medical treatment, 185 children (47.6%) were relieved of their symptoms, whereas persistent type B was observed in 203 children (52.4%) (Table 1).

**Table 1:** Sociodemographic and other parameters of participants Data are presented as numbers and percentages.

Variables		Frequency N (%)
Gender	Male	170 (43.8%)
	Female	218 (56.2%)
Tympanometry	Unilateral type B	138 (35.6%)
	Bilateral type B	250 (64.4%)
After 3 months of watchful waiting with follow-up±medical treatment	Relieved	185 (47.6%)
	Persist type B	203 (52.4%)

Table 2 shows the preoperative assessments and previous medical histories of the patients. Most of the cases were newly diagnosed (n=193, 95.07%), with only a small number being revised cases (n=10, 4.92%). Preoperative assessments revealed high incidences of allergic rhinitis (n=188, 92.61%) and adenoid enlargement (n=176, 86.70%), and adenotonsillar enlargement was present in 139 children (68.47%). In terms of middle ear conditions, secretory otitis media (SOM) was predominantly bilateral (n=160, 78.82%) compared to unilateral (n=39, 19.21%). Adhesive otitis media was relatively rare, with cases mainly bilateral (n=3, 1.48%) and only one unilateral case (0.49%). Previous surgical interventions were primarily myringotomy (n=9, 4.44%), with fewer cases of myringotomy with tube insertion (n=3, 1.48%).

**Table 2:** Preoperative assessment of patients and previous history Data are presented as numbers and percentages. OM: otitis media

Variables			Frequency N (%)
Case type	New		193 (95.07%)
	Revision		10 (4.92%)
Preoperative assessment	Adenoid		176 (86.70%)
	Adeno-tonsillar		139 (68.47%)
	Allergic rhinitis		188 (92.61%)
Persistent type B	Secretory OM	Unilateral	39 (19.21%)
		Bilateral	160 (78.82%)
	Adhesive OM	Unilateral	1 (0.49%)
		Bilateral	3 (1.48%)
Previous surgery	Myringotomy only		9
	Myringotomy with tube insertion		3

Table 3 shows the types of surgeries performed on the patients, their outcomes, and associated complications. Some surgeries were performed using microscopy, while others were performed by endoscopy. A large proportion of surgeries were done bilaterally (n=163, 80.30%) compared to unilateral surgeries (n=40, 19.70%). Among the surgical procedures, 94 patients (46.31%) underwent myringotomy with Shah tube insertion, 78 (38.42%) had Shepard tube insertion, and 31 (15.27%) had myringotomy only. Adenotonsillectomy was the more common surgical combination, performed in 153 cases (75.37%), while adenoidectomy was done in 50 cases (24.63%). Complications were relatively rare, with only one case each of infection and persistent perforation (both 0.49%), seven cases of moderate pain (3.45%), and 11 cases of minimal bleeding (5.42%). Postoperative follow-up at 2 weeks, 3 months, 6 months, and 12 months showed that 179 patients (88.18%) were completely relieved of symptoms (tympanometry type A), 10 (4.93%) were partially relieved (tympanometry type C), which required the continuation of the medication course, and small numbers had persistent issues: six cases (2.97%) each had speech problems leading to speech therapy referral (type A) and recurrence of symptoms (type B), while two cases (0.99%) involved prolonged tube stay necessitating tube removal.

**Table 3:** Different types of surgeries patients underwent and their outcomes Data are presented as numbers and percentages.

Variables		Frequency N (%)
Surgery side	Unilateral	40 (19.70%)
	Bilateral	163 (80.30%)
Surgery type	Myringotomy only	31 (15.27%)
	Myringotomy with tube insertion (Shepard tube)	78 (38.42%)
	Myringotomy with tube insertion (Shah tube)	94 (46.31%)
Surgical combination	Surgical combination - adenoidectomy	50 (24.63%)
	Adenotonsillectomy	153 (75.37%)
Complications	Infection	1 (0.49%)
	Persistent perforation	1 (0.49%)
	Moderate pain	7 (3.45%)
	Minimal bleeding	11 (5.42%)
Postoperative follow-up results with tympanometry types	Type A/complete relieved	179 (88.18%)
	Type A/still speech problem	6 (2.96%)
	Type B recurrence	6 (2.96%)
	Type C/partial relieved	10 (4.93%)
	Long-stay tube	(0.99%)

## Discussion

AOM and OME are common in children, often leading to hearing loss and developmental delays [16]. VTs are used to manage persistent MEEs by draining fluid and reducing pressure, which can decrease the severity of rAOM. A study by Spaw et al. (2024) shows that tympanostomy tubes improve hearing and reduce the risk of long-term complications by ventilating the middle ear and promoting fluid drainage [17]. While VT offers temporary relief and can reduce AOM recurrence, it carries risks like eardrum perforation and conductive hearing loss. Similarly, Al Sheeyab et al. (2023) show that a VT may have complications itself, like otorrhea, eardrum atrophy, perforation, serous otitis media, and tympanosclerosis [18]. Our study aimed to investigate the effect of the VT insertion procedure in children under 12 years old and its potential to restore normal hearing.

Notably, our study displays a significant prevalence of otitis media with particular demographic and clinical characteristics. The gender distribution, predominantly female (62.1%), is slightly unusual for otitis media, which typically shows no significant gender bias in most studies. Similarly, Mukara et al. (2017) show no significant gender differences in the prevalence of middle ear infections [19]. However, this could be reflective of the specific population and healthcare-seeking behavior in this region.

Moreover, the tympanometry results revealed that 64.4% of participants had bilateral type B findings, indicative of a fluid presence in the middle ear without signs of acute infection, significantly higher than unilateral type B, which stood at 35.6%. This suggests a high prevalence of OME among the children studied, which is consistent with literature indicating OME as a common condition in pediatric populations, particularly those between 2 and 12 years. Similarly, previous studies show that between 80% and 90% of all children will have otitis media with an effusion before school age [16,20,21]. The treatment within three months showed a nearly split outcome, with 47.6% relieved and 52.4% persisting, which emphasizes the challenging nature of managing OME and its tendency to become a recurring issue. Lanphear et al. (1997) show that recurrent otitis in preschool children rose from 18.7% to 26% in the last seven years, with infants experiencing the largest increase (OR=1.9, CI=1.3-2.9) [22].

Moreover, the high rate of adenoid hypertrophy (86.70%) and allergic rhinitis (92.61%) in our study highlights the well-documented association between these conditions and otitis media, particularly the non-suppurative forms like OME. Similarly, Mashat et al. (2022) found a correlation between adenoid hypertrophy and OME, which contributed to silent, progressive hearing loss in children [23]. Adenotonsillar hypertrophy being prevalent in 68.47% of the cases also underscores the common etiological linkage with Eustachian tube dysfunction, which promotes fluid retention and infection in the middle ear.

Interestingly, the majority of our cases were new diagnoses (95.07%), with minimal revisions (5.42%), suggesting effective initial management or possibly underreporting of recurrent cases. Our findings on secretory and adhesive otitis media further elaborate the complexity of otitis media presentations in this population, with a significant predilection for bilateral conditions, which are often more challenging to manage and have higher risks for prolonged hearing loss.

Notably, the surgical data shows a predominant reliance on bilateral interventions (80.30%), which is reflective of the bilateral nature of the disease in most children. A study by Uitti et al. (2013) shows that bilateral AOM is considered more severe than unilateral AOM, and several guidelines recommend more active treatment and/or follow-up of bilateral AOM [24]. The use of different tube types, namely Shepard and Shah, with a prevalence of 38.42% and 46.31%, respectively, showcases the tailored approach to managing varying severities and anatomical challenges in pediatric otitis media. Ahmad et al. (2016) show that VT treatment is recommended for children with OME. Parents often report that children talk better, hear better, are less irritable, sleep better, and behave better after myringotomy with the insertion of ear tubes [25]. The higher utilization of adenotonsillectomy (75.37%) compared to adenoidectomy alone (24.63%) could be indicative of the more aggressive approach taken in cases with significant adenotonsillar contributions to ear pathologies.

The complication rates were notably low, with infection and persistent perforation each at 0.49%, which is significantly lower, suggesting either an exceptionally skilled surgical technique or potentially underreported complications. Similarly, a study by Vahedian et al. (2020) showed that 98% of VT insertions caused otorrhea, with other complications including tympanosclerosis (14.1%), eardrum atrophy (6.8%), perforation (1.5%), and serous otitis recurrence (14.1%) [26]. The high success rate seen in tympanometry post-operation (88.18% complete relief) is encouraging and aligns with recent studies showing improved outcomes with comprehensive surgical strategies that address all underlying anatomical and pathological factors. Similarly, Otsuka et al. (2022) and Saraf et al. (2023) showed a 51.7% survival rate of VT at full-term placement and reported an improvement in quality of life in children [27,28].

Limitations

Several limitations of our study include a lack of a control group, which could provide comparative insights into VT efficacy. Additionally, the sample size may not represent all demographic groups equally, limiting generalizability. Retrospective data collection may also introduce recall biases, and the short follow-up period may not adequately capture long-term outcomes or recurrent complications. Lastly, the study is confined to a single medical center, which may influence the findings due to specific clinical practices or patient populations.

Implications and future direction

This study underscores the importance of careful monitoring for complications following VT insertion and suggests the need for alternative strategies to manage otitis media more effectively. It also highlights the potential benefits of combining VT with other surgical approaches to reduce recurrent otitis media incidences. Future research should focus on longitudinal studies to assess long-term outcomes of VT treatments across diverse populations. Implementing randomized controlled trials could provide more definitive evidence of effectiveness and safety. Additionally, exploring alternative and less invasive treatments could offer valuable insights into preventing recurrent episodes and reducing the reliance on surgical interventions in young children.

## Conclusions

Our study provides valuable insights into the epidemiology and management outcomes of OME among Saudi children. The high rates of successful surgical outcomes suggest effective surgical strategies, despite the persistent nature of some cases and/or occurrence of complications, indicating areas for potential improvement. Future research should aim to explore the impacts of preventive strategies, as well as the long-term auditory and developmental outcomes for these children.
